# Long-Chain Fatty Acids as Drivers of Neuroinflammation in Neurodegeneration: Mechanistic Links to Lipid Peroxidation, Ferroptosis, and Mitochondrial Dysfunction

**DOI:** 10.3390/nu18091392

**Published:** 2026-04-28

**Authors:** Rafail C. Christodoulou, Laura Lorentzen, Daniel Eller, Evros Vassiliou

**Affiliations:** 1 Division of Neuroimaging and Neurointervention, Department of Radiology, Stanford University, Stanford, CA 94304, USA; rafail99@stanford.edu (R.C.C.); deller@stanford.edu (D.E.); 2 Department of Biological Sciences, Kean University, Union, NJ 07083, USA; llorentz@kean.edu

**Keywords:** neuroinflammation, immunometabolism, long-chain fatty acids, astrogliosis, microglia, lipid peroxidation, oxidative stress, neurodegeneration

## Abstract

**Background:** Neurodegenerative diseases (NDs) are mainly considered disorders marked by severe immunometabolic imbalance, characterized by ongoing neuroinflammation and glial activation. While mitochondrial dysfunction and oxidative stress are well-known features, the upstream metabolic factors linking these pathological processes remain poorly understood. **Methods:** In this review, we examined recent preclinical and clinical studies exploring the connections between lipid metabolism, glial immunometabolism, and regulated cell death pathways. Our focus was on how long-chain fatty acids (LCFAs) facilitate communication among mitochondria, reactive oxygen species (ROS), and ferroptosis in Alzheimer’s disease (AD), Parkinson’s disease (PD), and amyotrophic lateral sclerosis (ALS). **Results**: New evidence shifts LCFAs from merely being passive indicators of cellular damage to active, upstream regulators of the neuroimmune response. Existing research shows that excess LCFA intake can overload astrocytic mitochondrial oxidative phosphorylation, leading to abnormal lipid droplet buildup and reactive astrogliosis. This lipid-driven reactivity promotes microglial polarization toward a persistent pro-inflammatory state. Notably, high levels of specific LCFAs, especially arachidonic acid, increase ROS production and lipid peroxidation. This lipotoxic environment ultimately triggers ferroptosis, an iron-dependent form of cell death shared across multiple NDs. **Conclusions:** The harmful interaction among mitochondrial dysfunction, lipid peroxidation, and ferroptosis is driven by an imbalance in LCFA levels. Addressing current challenges, such as the complex effects of polyunsaturated fatty acid supplementation, requires advanced techniques like single-cell multi-omics and artificial intelligence. Understanding this intricate lipidomic-transcriptomic crosstalk is crucial for moving toward personalized neuroimmunometabolism and developing new treatments to prevent ferroptosis.

## 1. Introduction

Neurodegenerative diseases (NDs), such as Alzheimer’s disease (AD), Parkinson’s disease (PD), and amyotrophic lateral sclerosis (ALS), are increasingly burdening global health due to aging populations [1]. These devastating disorders primarily involve progressive neuronal loss, leading to significant cognitive and motor decline [1,2]. Despite their high prevalence and societal and economic impact, current treatments are largely symptomatic. For instance, treatments for PD only temporarily relieve motor symptoms, do not stop disease progression, and often cause notable side effects over time [2]. These scenarios highlight the urgent need to understand the fundamental molecular causes of neuronal death. Recent findings suggest that neuroinflammation and oxidative stress are not just secondary effects but central, interconnected mechanisms that actively contribute to the progression of various NDs [1,3].

The central nervous system is particularly susceptible to metabolic and inflammatory damage due to its unique composition and high energy demands [4]. Besides adipose tissue, the brain has the highest lipid concentration in the human body, with lipids accounting for more than 50% of its dry weight [5]. These lipids are not just passive structural elements; they are vital for preserving neuronal membrane integrity, regulating synaptic functions, and supporting memory processes such as long-term potentiation [5,6]. Additionally, the active breakdown of specific membrane phospholipids generates essential lipid messengers that modulate cellular protection, repair, and survival signals [6]. Because of their deep dependence on precise lipid balance for normal function, neurons and glial cells are highly sensitive to disruptions or age-related changes in lipid metabolism [4].

Long-chain fatty acids (LCFAs) and polyunsaturated fatty acids (LC-PUFAs) are among the various lipid types in the brain [7]. They serve a crucial dual function in neurological health and disease. Essential LC-PUFAs like docosahexaenoic acid (DHA), an omega-3, and arachidonic acid (ARA), an omega-6, are incorporated into cell membranes, where they are essential for signal transduction, gene expression, and the maintenance of structural integrity [5]. However, when lipid balance is disturbed, LCFAs may become harmful drivers. For example, palmitic acid, the most common saturated fatty acid, can induce cellular stress and trigger robust pro-inflammatory responses in microglia [8]. Since LCFAs are poorly soluble, they depend on specialized intracellular chaperones like fatty acid-binding proteins (FABPs) for cell transport [9]. When these transport mechanisms are overwhelmed, unbound LCFAs accumulate within the cell [9]. This excessive accumulation leads to lipotoxicity, a harmful condition that causes oxidative stress, promotes neuroinflammation, and activates neurodegenerative processes [8,9].

The shift from physiological lipid metabolism to harmful neurotoxicity primarily involves glial cell immunometabolism and mitochondrial failure [10]. In the brain, astrocytes rely on mitochondrial oxidative phosphorylation to metabolize fatty acids and maintain lipid homeostasis [11]. When excessive fatty acids surpass this capacity, lipid droplet buildup occurs, increasing acetyl-CoA levels and triggering astrocyte reactivity. These reactive, lipid-rich astrocytes then promote oxidative stress in neurons and activate microglia [11]. Simultaneously, microglia undergo polarization that is tightly controlled by lipid metabolic reprogramming [12]. The buildup of ceramides and oxidized lipids drives microglia into a persistent pro-inflammatory state, worsening neuronal damage [12]. The combination of mitochondrial dysfunction, induced neuroinflammation, and disrupted lipid metabolism leads to a significant accumulation of lipid peroxides [13]. When combined with cellular iron buildup, this severe lipid peroxidation induces ferroptosis, which has recently been recognized as a key cause of neuronal death in various NDs [13,14].

While neuroinflammation, mitochondrial dysfunction, and ferroptosis are increasingly recognized in neurodegenerative diseases, most studies assess these markers separately. As disease development involves complex interactions among these molecular pathways, there is a pressing need to understand how they intersect [13,15]. In particular, the role of LCFAs as key upstream metabolic drivers linking these mechanisms remains to be fully elucidated. This review aims to synthesize existing evidence on the relationship between LCFAs, neuroinflammation, and neurodegeneration. It emphasizes how LCFA imbalance influences mitochondrial dysfunction, lipid peroxidation from oxidative stress, and ferroptosis, offering a pathway to identify new therapeutic targets for neuronal death. It is worth reiterating, however, that specific unsaturated LCFAs, particularly long-chain polyunsaturated fatty acids (LC-PUFAs) such as DHA and EPA, exhibit neuroprotective effects. Rather than driving neurodegeneration, DHA actively suppresses amyloid-beta-induced reactive oxygen species (ROS) in microglia and upregulates antioxidant pathways [16]. Additionally, omega-3 delivery preserves metabolic homeostasis in immune cells within the central nervous system, thereby mitigating neuroinflammation [17]. Monounsaturated fatty acids also offer protection. Recent lipidome analyses link higher circulating oleic acid to a reduced risk of mild cognitive impairment progressing to Alzheimer’s disease [18]. Ultimately, neurodegenerative progression is likely driven not merely by absolute LCFA overload, but by a critical imbalance between neurotoxic saturated species and these neuroprotective unsaturated species.

## 2. Literature Search Strategy

A structured literature review was created on 19 March 2026, in collaboration with an academic medical librarian, to identify studies on LCFAs and their role in neuroinflammation and neurodegenerative diseases. The search strategy adhered to recommended reporting standards for narrative reviews, prioritizing transparency in how the literature was identified and studies selected, while noting the non-systematic nature of narrative reviews [19,20]. Electronic databases such as PubMed/MEDLINE, Embase, and Scopus were searched to find relevant experimental and translational research. The search combined controlled vocabulary terms with free-text keywords related to LCFAs, lipid metabolism, neurodegenerative diseases, neuroinflammatory mechanisms, and pathways, including ferroptosis, lipid peroxidation, and mitochondrial dysfunction. Lipid terms included long-chain fatty acids, saturated and polyunsaturated fatty acids, palmitic acid, oleic acid, arachidonic acid, DHA, and EPA. Disease terms covered Alzheimer’s, Parkinson’s, ALS, Multiple sclerosis, Huntington’s, dementia, and general neurodegeneration. The mechanistic search included neuroinflammation, microglia activation, astrocyte responses, cytokines, inflammasomes, immunometabolism, TLR4 signaling, ferroptosis, lipid peroxidation, and mitochondrial dysfunction.

Searches were limited to English-language publications from 2020 onward (with notable exceptions made for seminal foundational studies) to focus on recent mechanistic insights and emerging ideas in lipid-related neurodegeneration. Case reports, editorials, and studies without mechanistic or biological relevance were excluded. Titles and abstracts were reviewed to identify studies on LCFA-associated neurobiological mechanisms, with an emphasis on research on inflammatory signaling, oxidative stress pathways, ferroptosis, mitochondrial dysfunction, and lipotoxicity in the central nervous system.

To supplement database searches, additional relevant studies were identified through citation tracking of key articles, examination of the references of highly relevant publications, and targeted searches for recent high-impact studies on emerging mechanistic ideas. This approach helped include conceptually important studies that might not have been captured by database searches alone.

A narrative review rather than a systematic review was conducted; this search strategy aimed to ensure broad conceptual and mechanistic coverage rather than exhaustive collection. While this approach can introduce selection bias, a recognized limitation of narrative reviews, efforts were made to maintain transparency in search design, inclusion criteria, and the thematic organization of the literature [21].

Full database-specific search strategies and Boolean logic are provided in Appendix A.

## 3. Discussion

### 3.1. Mechanistic Synthesis: LCFAs as Upstream Drivers of Neurodegeneration

LCFAs are increasingly recognized not only as passive markers of cellular damage but also as key upstream regulators of ND development [1]. Saturated LCFAs, such as palmitic acid, activate the TLR4 signaling pathway, leading to NF-κB translocation to the nucleus and pro-inflammatory cytokine production [22]. Furthermore, NF-κB-mediated transcription leads to an elevation of the NLRP3 sensor protein. Subsequent release of IL-1β reduces tight junction proteins, specifically Claudin-5 and Occludin, leading to increased blood-brain barrier permeability [23,24]. Modulation of the toxicity of saturated LCFA can be achieved through DHA and EPA supplementation or intermittent fasting, which aids in the clearance of excessive LCFAs and the augmentation of anti-peroxidation mechanisms [25]. Normally, the brain relies heavily on astrocytic mitochondrial oxidative phosphorylation to metabolize fatty acids and maintain lipid homeostasis [11]. However, when the fatty acid load surpasses the capacity of astrocytic oxidative phosphorylation, it triggers a significant pathophysiological change. Abnormal lipid metabolism causes lipid droplet accumulation and elevated acetyl-CoA levels, thereby increasing STAT3 acetylation and leading to robust reactive astrogliosis. This lipid-driven astrocyte reactivity acts as an upstream event that promotes neuronal fatty acid oxidation and oxidative stress, while also activating microglia via interleukin-3 signaling [11]. Additionally, this metabolic failure in astrocytes hampers the production of fatty acids and phospholipids that are crucial for myelin repair [11]. Because fatty acids influence neuroinflammation, immune responses, and blood-brain barrier function, their dysregulation is a primary trigger of neuroinflammation, oxidative stress, and progressive neuronal loss [1,11]. A summary of the major LCFA, their mechanistic roles, and their relevance across neurodegenerative diseases is presented in Table 1.

### 3.2. Integrated Mechanistic Model

Physiologically, the controlled, enzymatic breakdown of LCFAs generates essential lipid messengers critical for cellular protection and synaptic repair. Conversely, pathological oxidative stress drives unregulated, non-enzymatic lipid peroxidation, producing toxic end-products like 4-hydroxynonenal (4-HNE), malondialdehyde (MDA), and carboxyethylpyrrole (CEP) [35,36,37]. Unlike transient physiological signals, these highly reactive products irreversibly bind to cellular macromolecules, severely disrupt neuronal membrane integrity, and act as the direct chemical executioners of ferroptotic cell death.

**Figure 1:**
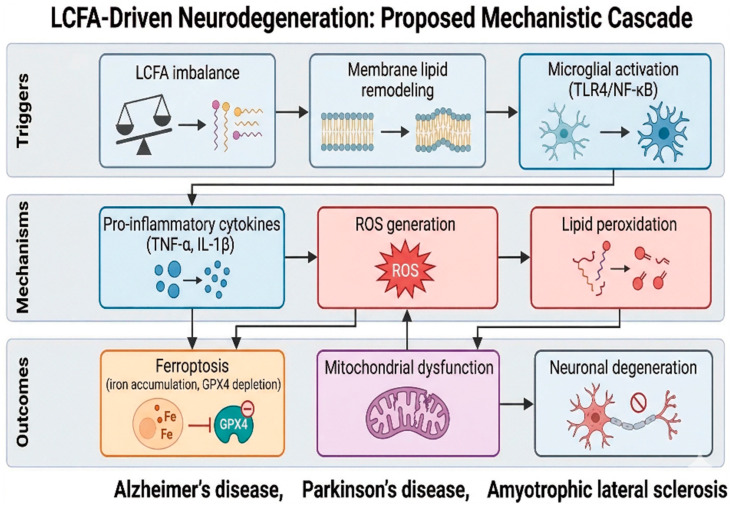
Proposed mechanistic cascade linking long-chain fatty acid (LCFA) dysregulation to neurodegeneration. LCFA imbalance promotes membrane lipid remodeling and microglial activation, leading to pro-inflammatory cytokine release, oxidative stress, and lipid peroxidation. These processes contribute to ferroptosis, mitochondrial dysfunction, and ultimately neuronal degeneration across neurodegenerative diseases. Created in BioRender. Christodoulou, R. (2026). https://BioRender.com/gq4vx4t (accessed on 26 April 2025).

**Figure 2:**
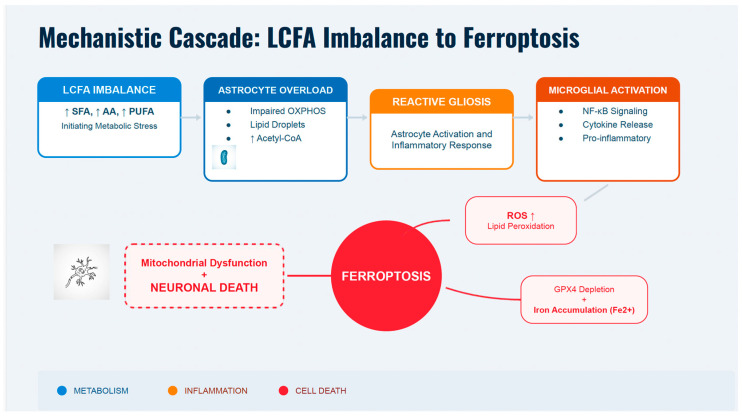
Mechanistic cascade linking LCFA imbalance to ferroptosis and neuronal death. Long-chain fatty acid (LCFA) imbalance, with increased saturated fatty acids (SFAs), arachidonic acid (AA), and polyunsaturated fatty acids (PUFAs), triggers metabolic stress in the CNS. Excess lipids overwhelm astrocyte mitochondria, causing lipid droplet buildup and higher acetyl-CoA levels, promoting reactive astrogliosis. This worsens pro-inflammatory signaling and activates microglia, leading to cytokine release and neuroinflammation via NF-κB. Simultaneously, ROS production, lipid peroxidation, iron accumulation (Fe^2+^), and glutathione peroxidase 4 (GPX4) depletion induce ferroptosis. Mitochondrial dysfunction worsens oxidative stress, creating a cycle that results in neuronal death. This cascade links metabolic dysregulation, inflammation, and cell death in neurodegenerative diseases.

While ferroptosis is often considered a late-stage phenomenon leading to neuronal death, the biochemical events that precede it occur at much earlier stages. During these initial phases, lipid peroxidation is initiated under heightened oxidative stress, largely driven by an LCFA imbalance. Cells rely on specific localized defenses against lipid peroxidation. The primary enzymatic guardian is Glutathione Peroxidase 4 (GPX4), which uniquely reduces toxic, membrane-bound lipid hydroperoxides to inert alcohols, using endogenous glutathione as an essential cofactor [38]. Synergistically, alpha-tocopherol (Vitamin E) acts as a lipophilic, chain-breaking antioxidant within the bilayer, directly intercepting peroxyl radicals [39]. Ferroptosis initiates only when accumulating oxidized LCFAs overwhelm these specific defenses. At these early stages, GPX4 provides substantial protection. However, the accumulation of saturated LCFAs shifts the equilibrium toward higher concentrations of ROS and worsening mitochondrial dysfunction. As mitochondrial impairment persists, oxidative stress is further amplified in a self-perpetuating cycle. In conjunction with increased levels of Fe^2+^ resulting from blood-brain barrier permeability deterioration and substantial increases in ROS, the pathological cascade reaches a tipping point characterized by the uncontrolled peroxidation of LC-PUFAs and, ultimately, ferroptosis. This metabolic track is a connected series of mechanisms where LCFA imbalance initiates microglial activation, ultimately leading to ferroptosis and mitochondrial failure. The abnormal release of certain LCFAs actively causes microglial dysfunction. Notably, increased free ARA from phospholipids increases cellular reactive oxygen species (ROS) and lipid peroxidation in microglia [26]. This oxidative stress from LCFAs is further exacerbated by the production of pro-ferroptotic polyunsaturated fatty acids (PUFAs), which damage mitochondrial health, reduce the activity of the protective enzyme glutathione peroxidase 4 (GPX4), and induce high mitochondrial ROS [27]. As lipid peroxides accumulate in microglial and neuronal membranes in the presence of iron, they trigger ferroptosis, an emerging form of cell death linked to neurodegeneration. The connections among lipid metabolism, inflammation, and cell death are closely intertwined. For instance, certain linoleic acid derivatives can influence microglial activation by simultaneously regulating NF-κB inflammatory signaling and ferroptotic pathways [28]. Evidence suggests that the LCFA imbalance and ARA release activate microglia, leading to excessive ROS and uncontrolled lipid peroxidation [26,40]. This harmful lipid buildup eventually causes severe mitochondrial damage, including loss of membrane potential, leading to neuronal death via ferroptosis (Figure 1 and Figure 2) [27,40]. When long-chain fatty acids (LCFAs), particularly palmitic acid, exceed mitochondrial β-oxidation capacity, they accumulate and uncouple the electron transport chain, collapsing the membrane potential (ΔΨ_m_). This lipotoxic stress, in conjunction with elevated ROS and calcium dysregulation, triggers the opening of the mitochondrial permeability transition pore (mPTP), a complex inhibited by cyclosporine A. Persistent mPTP opening induces mitochondrial swelling, rupture, and profound oxidative stress. The massive efflux of superoxide radicals drives lipid peroxidation of neuronal polyunsaturated fatty acids (LC-PUFAs). The existing iron imbalance and the unchecked accumulation of lipid peroxides act as the primary initiation signals for ferroptosis.

### 3.3. Disease Convergence: Shared Mechanisms Across Disorders

The significant role of LCFA dysregulation provides a unifying mechanistic framework explaining the commonalities observed across different NDs, such as AD, PD, and ALS. Although these diseases present with distinct clinical features and primary protein deposits, they share fundamental lipid-related pathologies [1]. Brain metabolomics comparisons demonstrate that various NDs exhibit notably similar metabolic changes, highlighting a metabolic vulnerability among tauopathies [41]. In ALS, progressive motor neuron loss is closely associated with notable changes in lipid categories, including fatty acids and sphingolipids, which contribute to excitotoxicity, mitochondrial impairment, and neuroimmune responses [42]. Similarly, pathology in PD extends beyond α-synuclein aggregation to encompass systemic, multifaceted mechanisms in which metabolic issues, particularly lipid metabolism, and neuroinflammation are central [43]. Across these disorders, lipid peroxide formation and ferroptosis serve as common drivers of neuronal death [27]. Therefore, neuroinflammation, oxidative stress, and LCFA-mediated ferroptosis constitute interconnected, universal mechanisms that link diverse NDs [1,27,42,43].

### 3.4. Clinical and Translational Implications

Recognizing LCFAs as upstream drivers of neurodegeneration opens significant translational opportunities for biomarker identification, dietary strategies, and new drug targets [2,12,44,45]. Dietary approaches, such as supplementing with PUFAs, especially omega-3s, are gaining clinical attention as key metabolic interventions [29,30,46]. Omega-3 PUFAs directly compete with pro-inflammatory omega-6 fatty acids such as arachidonic acid, thereby reducing microglial activation, boosting neurotrophic factors, and restoring mitochondrial membrane fluidity in PD and other NDs [29,30,31]. Different omega-3 derivatives have distinct cellular effects. Eicosapentaenoic acid (EPA) has been shown to be more effective than docosahexaenoic acid (DHA) in enhancing mitochondrial membrane potential and maintaining cardiolipin integrity [32,34]. This competitive mechanism underpins advanced nutraceutical strategies that link daily nutrition to neuroprotection [2,15,46,47,48]. Clinically, comprehensive diets, such as those high in omega-3 PUFAs and low in pro-inflammatory omega-6 and saturated fats, are being tested for their potential to mitigate oxidative stress and mitochondrial dysfunction in conditions such as ALS and neuroinflammatory disorders with neurodegenerative overlap, such as multiple sclerosis (MS) [49,50,51,52,53]. Metabolic reprogramming via intermittent fasting shows promise in modulating the neuroimmune environment by promoting fatty acid oxidation and mitochondrial clearance [54]. Beyond lipids, the ferroptotic cascade offers a critical pharmacological target. New ferroptosis inhibitors, glutathione peroxidase 4 (GPX4) modulators, and natural plant compounds are under investigation for their ability to cross the blood-brain barrier, reconfigure oxidative stress pathways, and directly inhibit lipid peroxidation in neurons [13,15,55,56,57,58,59]. Simultaneously, upstream strategies aim to prevent harmful LCFA accumulation and ferroptosis by targeting lipid transport and activation, such as inhibitors of fatty acid-binding proteins (FABP) or acyl-CoA synthetase long-chain family member 4 (ACSL4), which could stop intracellular LCFA buildup before irreversible mitochondrial failure ensues [9,60,61]. Multi-target agents such as (benzo)thiazine derivatives are also promising for regulating lipid profiles and providing direct neuroprotection against oxidative damage [62]. A meaningful laboratory biomarker approach for NDs should go beyond single-analyte tests and instead combine markers of LCFA dysregulation, oxidative lipid modification, mitochondrial stress, and ferroptosis susceptibility. Key upstream lipid markers include palmitic acid, ARA, DHA, EPA, and the omega-6/omega-3 imbalance, as current evidence indicates that neurodegeneration is more influenced by a harmful shift toward pro-inflammatory and pro-oxidant lipid states than by LCFA quantity alone. Recent studies further support the biological relevance of ARA mobilization and peroxidation in AD, with abnormal ARA remodeling linked to microglial dysfunction and oxidative injury [26]. Downstream of LCFA imbalance, promising markers of oxidative modification include lipid hydroperoxides, oxidized PUFA derivatives, and classical lipid peroxidation products such as 4-hydroxynonenal (4-HNE) and malondialdehyde (MDA), which remain widely used readouts of oxidative lipid injury and ferroptosis-related stress [63]. In ALS, carboxyethylpyrrole (CEP), a DHA peroxidation product generated under inflammation-dependent oxidative stress, has been proposed as a particularly informative example of disease-linked oxidative lipid damage [64]. Markers of mitochondrial dysfunction should also be considered, including ROS excess, impaired fatty-acid oxidation, acylcarnitine abnormalities, cardiolipin remodeling, and circulating mitochondrial DNA-related measures, although these currently remain more promising translational biomarkers than routine clinical assays [65]. Finally, ferroptosis-oriented laboratory profiling may include GPX4 depletion or loss of activity, ACSL4-associated lipid remodeling, iron accumulation, and combined iron–lipid peroxidation signatures, which are increasingly recognized as central to neurodegenerative injury, particularly in AD [66].

**Figure 3:**
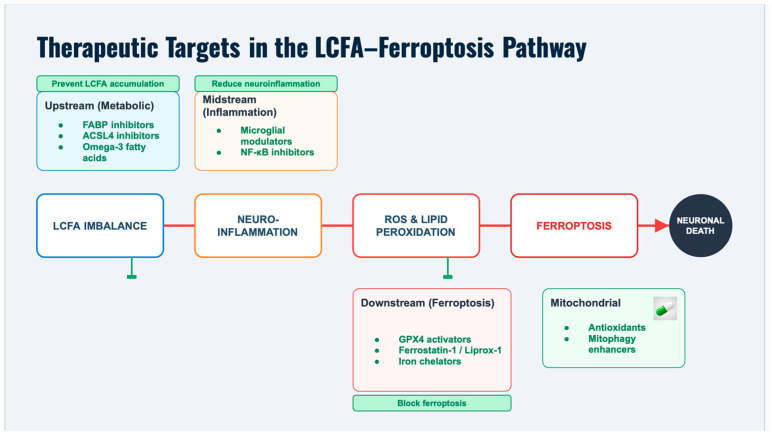
Therapeutic targets across the LCFA–ferroptosis axis in neurodegeneration. Intervention points along the LCFA-driven neurodegenerative cascade. Upstream strategies prevent LCFA buildup and lipotoxicity by modulating lipid transport, inhibiting FABPs and ACSL4, and dietary omega-3s. Midstream methods reduce neuroinflammation by targeting microglia and NF-κB pathways, thereby lowering cytokine levels and oxidative stress. Downstream actions inhibit ferroptosis via preservation of GPX4, ferroptosis inhibitors (e.g., ferrostatin-1, liproxstatin-1), and iron chelation to limit lipid peroxidation. Mitochondrial therapies, including antioxidants and mitophagy enhancers, restore redox balance and cell health.

Beyond these broad, mechanism-based strategies, several pharmacological and nutraceutical agents have been proposed as adjunctive approaches to address LCFA-related mitochondrial dysfunction, oxidative stress, and susceptibility to ferroptosis. Mechanistically, carnitine and acetyl-L-carnitine are promising because they support mitochondrial fatty acid transport and β-oxidation, which could lower LCFA buildup, enhance mitochondrial performance, and reduce oxidative damage; however, clinical data in neurodegenerative conditions remain mixed and are insufficient to support routine use [67]. Meldonium is also of interest, as it influences mitochondrial energy metabolism and has shown neuroprotective and mitochondria-preserving effects in preclinical models, including ischemic injury and Huntington’s disease, but its role in chronic neurodegeneration remains preliminary and largely preclinical [68]. Among antioxidants, alpha-tocopherol (vitamin E) has the strongest scientific basis here because it is a fat-soluble chain-breaking antioxidant that can limit lipid peroxidation and has been linked to inhibition of ferroptosis; notably, a randomized trial found slower functional decline with alpha-tocopherol in mild-to-moderate Alzheimer’s, though its broader efficacy across neurodegenerative diseases remains uncertain [69,70]. Glutathione is particularly relevant, as its depletion and reduced GPX4-dependent detoxification are key contributors to ferroptotic vulnerability; early Parkinson’s studies suggest that intranasal glutathione can boost central nervous system levels and is feasible, but definitive disease-modifying effects remain unproven [71,72]. Conversely, evidence for thiotriazoline appears to be mainly preclinical, focusing on oxidative stress and mitochondrial injury models, especially ischemic conditions, rather than on large clinical trials in major NDs [73]. These agents can be conceptually mapped onto the therapeutic framework shown in Figure 3, particularly within mitochondrial-supportive, antioxidant, and anti-ferroptotic intervention domains.

### 3.5. Knowledge Gaps

Despite these compelling mechanistic links, several critical knowledge gaps impede the immediate clinical translation of lipid-targeted therapies [9,54,74,75]. First, comprehensive lipidomic profiling in neurodegenerative diseases faces major technical challenges due to the immense structural heterogeneity of LCFA subtypes, membrane phospholipids, and dynamic lipid rafts within neural tissue [74,76,77]. It remains exceedingly difficult to temporally distinguish between the physiological mobilization of lipids for synaptic signaling and the pathological accumulation of lipotoxic droplets in living human brains [6,78]. Second, there is a pronounced lack of longitudinal human data tracking lipid dysregulation from prodromal to advanced disease stages [33,64,79].

Translating preclinical findings into human therapies is further complicated by severe discrepancies observed in standard animal models. While omega-3 supplementation is widely viewed as neuroprotective, striking experimental evidence demonstrates that Eicosapentaenoic Acid (EPA) administration accelerates disease progression and exacerbates motor neuron loss in a widely used transgenic mouse model of ALS [80]. Such paradoxical findings underscore a profound gap in our understanding of dose-dependent lipid toxicity and disease-specific microglial responses. Furthermore, the genetic regulation of these pathways remains incompletely mapped, and the precise mechanisms by which the apolipoprotein E4 (APOE4) risk allele specifically drives oxidative lipid metabolism and exacerbates ferroptosis in sporadic AD remain poorly understood [76]. Lastly, clinical translation is hindered by patient heterogeneity, limited blood-brain barrier penetration of antioxidants, and the lack of noninvasive in vivo biomarkers [81,82]. Consequently, these foundational mechanisms require continuous validation in humanized models.

While specific inflammation-dependent oxidative stress metabolites, such as carboxyethylpyrrole derivatives resulting from lipid peroxidation, have been identified as disease hallmarks in ALS, translating these findings into reliable plasma or neuroimaging biomarkers remains an unmet clinical need [64,83]. Accurately capturing the intricate, bidirectional crosstalk between oxidative stress and neuroinflammation requires the simultaneous integration of lipidomics and transcriptomics, a multi-omics approach that is currently limited in large-scale human cohorts due to high costs and constraints on brain tissue availability [77].

### 3.6. Future Research Directions

Future investigations must prioritize high-resolution, multi-omics techniques to fully decode the neuroimmunometabolism of the degenerating brain [77]. Single-nucleus RNA sequencing (snRNA-seq) in autosomal-dominant AD and risk-variant carriers has highlighted the absolute necessity of mapping lipid-driven transcriptomic changes at the single-cell level [84]. Expanding these platforms to incorporate spatial transcriptomics and advanced in situ lipidomics will enable researchers to visualize precisely where lipid droplets accumulate and which glial subpopulations initiate the ferroptotic cascade [60,77,85]. Additionally, exploring the gut-brain axis will be essential, as the microbiome plays a critical role in systemic lipid regulation and in the generation of neuroactive short- and medium-chain fatty acids [86].

To manage and interpret the immense, high-dimensional datasets generated by multi-omics profiling, integrating artificial intelligence (AI) and machine learning algorithms will be paramount. Advanced AI models could accelerate the discovery of complex lipid biomarker signatures, model the 3D structures of uncharacterized lipid-binding proteins for novel drug discovery, and accurately predict individualized disease trajectories. Ultimately, leveraging these AI-driven insights could pave the way for precision nutrition and highly individualized pharmacological interventions tailored to a patient’s unique lipidomic, metabolic, and genetic profile. Moreover, integrating lipidomics with AI-driven biomarker discovery may accelerate the identification of clinically relevant LCFA signatures.

Finally, future clinical trials must expand beyond passive supplementation to evaluate active metabolic interventions. Rigorous investigations into how targeted lifestyle modifications, such as structured physical exercise, can metabolically reprogram cellular iron handling and lipid oxidation to directly mitigate ferroptosis will be critical for developing holistic, disease-modifying strategies [87].

### 3.7. Limitations of the Review

Several limitations of this review should be recognized. Firstly, the narrative approach naturally introduces potential bias in selecting and weighing certain preclinical and clinical studies. Secondly, there is considerable methodological variation across the referenced literature, from isolated in vitro glial cultures to various transgenic animal models, which makes direct comparison of specific LCFA levels and ferroptotic triggers challenging. Lastly, the limited number of large-scale, long-term, randomized controlled clinical trials focusing on particular LCFA or ferroptosis-targeted therapies means that conclusive clinical recommendations cannot be made at present.

## 4. Conclusions

Recent research shifts the view of LCFAs from being mere passive biomarkers of cellular debris to active contributors to neurodegeneration. When the ability of astrocytes and microglia to manage lipids is overwhelmed, LCFAs can initiate a harmful cascade—leading to mitochondrial dysfunction, glial cell activation, and increased reactive oxygen species (ROS) production and lipid peroxidation. Such conditions promote ferroptosis, a form of iron-dependent cell death implicated in AD, PD, ALS, and MS. Recognizing this common lipid-related pathology offers new therapeutic possibilities, including dietary omega-3 intake, metabolic reprogramming, and ferroptosis inhibition. Supplementation with specific LC-PUFAs has so far been disappointing. A possible explanation may be the timing factor. Once substantial neurodegeneration has been established, it is impossible to reverse the pathology. Transport mechanisms might also play a role in limiting the delivery of LC-PUFAs (primarily DHA and EPA) to the brain, thereby limiting their therapeutic effects. There is evidence that APOE4 genotypes, which are associated with a higher risk of AD, are less capable of delivering DHA to the brain [88,89]. Furthermore, lysophosphatidylcholine-bound DHA (LPC-DHA) seems to be a much more effective delivery mechanism than purified specific fatty acids [90]. Levels of lysophosphatidylcholine-bound DHA (LPC-DHA) are significantly higher in whole-food sources than in purified supplements, thereby augmenting DHA transport efficiency to the central nervous system (CNS).

It is not surprising that the MIND diet (Mediterranean-DASH Intervention for Neurodegenerative Delay) is highly recommended by neurologists as a step toward delaying neurodegeneration [91]. A better understanding of the physiological and biochemical pathways involving LCFAs will aid in developing more effective prevention strategies. However, implementing these strategies requires a nuanced understanding of lipid diversity and dose effects to avoid overly simplistic solutions. Future studies should leverage single-cell multi-omics and AI to explore cell-specific lipid-gene interactions, paving the way for personalized treatments that can prevent irreversible neuronal damage.

## Figures and Tables

**Table 1:** LCFA mechanisms across neurodegenerative diseases.

LCFA Type	Mechanism	Cellular Effect	Disease Relevance	Key Reference
**Palmitic acid (Saturated LCFA)**	Causes cellular stress	Triggers strong pro-inflammatory responses in microglia	General neurodegeneration and lipotoxicity	[8]
**Arachidonic acid (ARA/Omega-6)**	Increases cellular reactive oxygen species (ROS) and lipid peroxidation	Promotes microglial dysfunction	General neurodegeneration and Aβ pathology	[26]
**Pro-ferroptotic PUFAs**	Damages mitochondrial health and reduces glutathione peroxidase 4 (GPX4) activity	Induces high mitochondrial ROS and triggers ferroptosis	General neurodegeneration	[27]
**Linoleic acid derivatives (e.g., α-dimorphecolic acid)**	Simultaneously regulates NF-κB inflammatory signaling and ferroptotic pathways	Influences and suppresses microglial activation	Neuroinflammation	[28]
**Omega-3 PUFAs**	Competes with pro-inflammatory omega-6 fatty acids	Reduces microglial activation, boosts neurotrophic factors, and restores mitochondrial membrane fluidity	Parkinson’s disease (PD) and other NDs	[29,30,31]
**Eicosapentaenoic acid (EPA/Omega-3)**	Enhances mitochondrial membrane potential and maintains cardiolipin integrity; paradoxically accelerates disease progression in specific models	Distinct cellular effects compared to DHA; can exacerbate motor neuron loss	Amyotrophic lateral sclerosis (ALS)	[32,33]
**Docosahexaenoic acid (DHA/Omega-3)**	Metabolites of DHA (Neuroprotectin D1) protect against amyloid beta 42 neurotoxicity via induction of antiapoptotic genes bcl-2, bcl-xl, bfl-1	Protection against amyloid beta 42 neurotoxicity	Alzheimer Disease (AD)	[34]

## Data Availability

No new data were created or analyzed in this study.

## Appendix A

### Appendix A.1. PubMed Search

**Set #**	**Concept**	**Syntax**	**Results**
1	Long-Chain Fatty Acids/Lipid Metabolism	long-chain fatty acids OR LCFA OR saturated fatty acids OR polyunsaturated fatty acids OR monounsaturated fatty acids OR PUFA OR lipid metabolism OR fatty acid metabolism OR lipid dysregulation OR lipid homeostasis OR lipotoxicity OR palmitic acid OR oleic acid OR arachidonic acid OR docosahexaenoic acid OR DHA OR eicosapentaenoic acid OR EPA	
2	Neurodegenerative Diseases	neurodegeneration OR neurodegenerative diseases OR neurodegenerative disorders OR Alzheimer disease OR Parkinson disease OR amyotrophic lateral sclerosis OR ALS OR multiple sclerosis OR Huntington disease OR dementia	
3	Neuroinflammation/Immune Mechanisms	neuroinflammation OR neuroimmune OR microglia OR microglial activation OR astrocytes OR cytokines OR chemokines OR inflammasome OR NLRP3 inflammasome OR immune response OR innate immunity OR immunometabolism OR TLR4 signaling	
4	Mechanistic Pathways	ferroptosis OR lipid peroxidation OR mitochondrial dysfunction	
5	Limits/Filters	2020/01/01:3000/12/31[Date–Publication] AND english[Language] NOT (case reports[Publication Type] OR editorial[Publication Type] OR case reports[Title] OR case series[Title/Abstract])	
6	Full combined query with Limits/Filters run on 19 March 2026	((“long chain fatty acid*”[Title/Abstract] OR “LCFA”[Title/Abstract] OR “saturated fatty acid*”[Title/Abstract] OR “polyunsaturated fatty acid*”[Title/Abstract] OR “monounsaturated fatty acid*”[Title/Abstract] OR “PUFA”[Title/Abstract] OR “lipid metabolism”[Title/Abstract] OR “fatty acid metabolism”[Title/Abstract] OR “lipid dysregulation”[Title/Abstract] OR “lipid homeostasis”[Title/Abstract] OR “lipotoxicity”[Title/Abstract] OR “palmitic acid”[Title/Abstract] OR “oleic acid”[Title/Abstract] OR “arachidonic acid”[Title/Abstract] OR “docosahexaenoic acid”[Title/Abstract] OR “DHA”[Title/Abstract] OR “eicosapentaenoic acid”[Title/Abstract] OR “EPA”[Title/Abstract]) AND (“Neurodegenerative Diseases”[MeSH Terms] OR “alzheimer disease”[MeSH Terms] OR “parkinson disease”[MeSH Terms] OR “Amyotrophic Lateral Sclerosis”[MeSH Terms] OR “Multiple Sclerosis”[MeSH Terms] OR “huntington disease”[MeSH Terms] OR “Dementia”[MeSH Terms] OR “neurodegeneration”[Title/Abstract] OR “neurodegenerative disease*”[Title/Abstract] OR “neurodegenerative disorder*”[Title/Abstract] OR “alzheimer disease”[Title/Abstract] OR “parkinson disease”[Title/Abstract] OR “Amyotrophic Lateral Sclerosis”[Title/Abstract] OR “ALS”[Title/Abstract] OR “Multiple Sclerosis”[Title/Abstract] OR “huntington disease”[Title/Abstract] OR “Dementia”[Title/Abstract]) AND (“Microglia”[MeSH Terms] OR “Astrocytes”[MeSH Terms] OR “Cytokines”[MeSH Terms] OR “Inflammasomes”[MeSH Terms] OR “Toll-Like Receptor 4”[MeSH Terms] OR “neuroinflamm*”[Title/Abstract] OR “neuroimmune”[Title/Abstract] OR “microglia*”[Title/Abstract] OR “microglial activation”[Title/Abstract] OR “astrocyte*”[Title/Abstract] OR “cytokine*”[Title/Abstract] OR “chemokine*”[Title/Abstract] OR “inflammasome*”[Title/Abstract] OR “NLRP3 inflammasome”[Title/Abstract] OR “immune response”[Title/Abstract] OR “innate immunity”[Title/Abstract] OR “immunometabolism”[Title/Abstract] OR “TLR4”[Title/Abstract] OR “TLR4 signaling”[Title/Abstract]) AND (“Ferroptosis”[MeSH Terms] OR “Lipid Peroxidation”[MeSH Terms] OR “mitochondrial dysfunction”[Title/Abstract] OR “Ferroptosis”[Title/Abstract] OR “Lipid Peroxidation”[Title/Abstract] OR “mitochondrial dysfunction”[Title/Abstract]) AND 2020/01/01:3000/12/31[Date–Publication] AND “english”[Language]) NOT (“case reports”[Publication Type] OR “Editorial”[Publication Type] OR “case reports”[Title] OR “case series”[Title/Abstract])	93

### Appendix A.2. Embase Search

**Set #**	**Concept**	**Syntax**	**Results**
1	Long-Chain Fatty Acids/Lipid Metabolism	long-chain fatty acids OR LCFA OR saturated fatty acids OR polyunsaturated fatty acids OR monounsaturated fatty acids OR PUFA OR lipid metabolism OR fatty acid metabolism OR lipid dysregulation OR lipid homeostasis OR lipotoxicity OR palmitic acid OR oleic acid OR arachidonic acid OR docosahexaenoic acid OR DHA OR eicosapentaenoic acid OR EPA	
2	Neurodegenerative Diseases	neurodegeneration OR neurodegenerative diseases OR neurodegenerative disorders OR Alzheimer disease OR Parkinson disease OR amyotrophic lateral sclerosis OR ALS OR multiple sclerosis OR Huntington disease OR dementia	
3	Neuroinflammation/Immune Mechanisms	neuroinflammation OR neuroimmune OR microglia OR microglial activation OR astrocytes OR cytokines OR chemokines OR inflammasome OR NLRP3 inflammasome OR immune response OR innate immunity OR immunometabolism OR TLR4 signaling	
4	Mechanistic Pathways	ferroptosis OR lipid peroxidation OR mitochondrial dysfunction	
5	Limits/Filters	[2020–2026]/py AND [english]/lim NOT (‘case report’/it OR ‘editorial’/it)	
6	Full combined query with Limits/Filters run on 19 March 2026	(‘long-chain fatty acid*’:ti,ab OR lcfa:ti,ab OR ‘saturated fatty acid*’:ti,ab OR ‘polyunsaturated fatty acid*’:ti,ab OR ‘monounsaturated fatty acid*’:ti,ab OR pufa:ti,ab OR ‘lipid metabolism’:ti,ab OR ‘fatty acid metabolism’:ti,ab OR ‘lipid dysregulation’:ti,ab OR ‘lipid homeostasis’:ti,ab OR lipotoxicity:ti,ab OR ‘palmitic acid’:ti,ab OR ‘oleic acid’:ti,ab OR ‘arachidonic acid’:ti,ab OR ‘docosahexaenoic acid’:ti,ab OR dha:ti,ab OR ‘eicosapentaenoic acid’:ti,ab OR epa:ti,ab) AND (‘neurodegenerative disease’/exp OR ‘neurodegenerative disease’ OR ‘alzheimer disease’/exp OR ‘alzheimer disease’ OR ‘parkinson disease’/exp OR ‘parkinson disease’ OR ‘amyotrophic lateral sclerosis’/exp OR ‘amyotrophic lateral sclerosis’ OR ‘multiple sclerosis’/exp OR ‘multiple sclerosis’ OR ‘huntington disease’/exp OR ‘huntington disease’ OR ‘dementia’/exp OR dementia OR neurodegeneration:ti,ab OR ‘neurodegenerative disease*’:ti,ab OR ‘neurodegenerative disorder*’:ti,ab OR ‘alzheimer disease’:ti,ab OR ‘parkinson disease’:ti,ab OR ‘amyotrophic lateral sclerosis’:ti,ab OR als:ti,ab OR ‘multiple sclerosis’:ti,ab OR ‘huntington disease’:ti,ab OR dementia:ti,ab) AND (‘neuroinflammation’/exp OR ‘neuroinflammation’ OR ‘microglia’/exp OR microglia OR ‘astrocyte’/exp OR astrocyte OR ‘cytokine’/exp OR cytokine OR ‘chemokine’/exp OR chemokine OR ‘inflammasome’/exp OR inflammasome OR ‘nlrp3 inflammasome’/exp OR ‘toll like receptor 4’/exp OR ‘toll like receptor 4’ OR neuroinflamm*:ti,ab OR neuroimmune:ti,ab OR microglia*:ti,ab OR ‘microglial activation’:ti,ab OR astrocyte*:ti,ab OR cytokine*:ti,ab OR chemokine*:ti,ab OR inflammasome*:ti,ab OR ‘nlrp3 inflammasome’:ti,ab OR ‘immune response’:ti,ab OR ‘innate immunity’:ti,ab OR immunometabolism:ti,ab OR tlr4:ti,ab OR ‘tlr4 signaling’:ti,ab) AND (‘ferroptosis’/exp OR ferroptosis OR ‘lipid peroxidation’/exp OR ‘lipid peroxidation’ OR ‘mitochondrial dysfunction’/exp OR ‘mitochondrial dysfunction’ OR ferroptosis:ti,ab OR ‘lipid peroxidation’:ti,ab OR ‘mitochondrial dysfunction’:ti,ab) AND [2020–2026]/py AND [english]/lim NOT (‘case report’/it OR ‘editorial’/it)	198

### Appendix A.3. Scopus Search

**Set #**	**Concept**	**Syntax**	**Results**
1	Long-Chain Fatty Acids/Lipid Metabolism	long-chain fatty acids OR LCFA OR saturated fatty acids OR polyunsaturated fatty acids OR monounsaturated fatty acids OR PUFA OR lipid metabolism OR fatty acid metabolism OR lipid dysregulation OR lipid homeostasis OR lipotoxicity OR palmitic acid OR oleic acid OR arachidonic acid OR docosahexaenoic acid OR DHA OR eicosapentaenoic acid OR EPA	
2	Neurodegenerative Diseases	neurodegeneration OR neurodegenerative diseases OR neurodegenerative disorders OR Alzheimer disease OR Parkinson disease OR amyotrophic lateral sclerosis OR ALS OR multiple sclerosis OR Huntington disease OR dementia	
3	Neuroinflammation/Immune Mechanisms	neuroinflammation OR neuroimmune OR microglia OR microglial activation OR astrocytes OR cytokines OR chemokines OR inflammasome OR NLRP3 inflammasome OR immune response OR innate immunity OR immunometabolism OR TLR4 signaling	
4	Mechanistic Pathways	ferroptosis OR lipid peroxidation OR mitochondrial dysfunction	
5	Limits/Filters	(PUBYEAR > 2019 AND PUBYEAR < 2027) AND (LIMIT-TO (LANGUAGE, ‘English’))	
6	Full combined query with Limits/Filters run on 3/19/2026	TITLE-ABS-KEY (“long-chain fatty acid*” OR lcfa OR “palmitic acid” OR “oleic acid” OR “arachidonic acid” OR “docosahexaenoic acid” OR dha OR “eicosapentaenoic acid” OR epa) AND TITLE-ABS-KEY (neurodegeneration OR “neurodegenerative disease*” OR “neurodegenerative disorder*” OR Alzheimer* OR Parkinson* OR “amyotrophic lateral sclerosis” OR als OR “multiple sclerosis” OR Huntington* OR dementia) AND TITLE-ABS-KEY (neuroinflamm* OR microglia* OR astrocyte* OR inflammasome* OR nlrp3 OR immunometabolism OR tlr4) AND TITLE-ABS-KEY (ferroptosis OR “lipid peroxidation” OR “mitochondrial dysfunction”) AND PUBYEAR > 2019 AND PUBYEAR < 2027	81
